# Non-charismatic waterbodies and ecosystem disservices: Mine pit lakes are underrepresented in the literature

**DOI:** 10.3389/fmicb.2022.1063594

**Published:** 2022-11-29

**Authors:** Rachele Bernasconi, Mark A. Lund, Melanie L. Blanchette

**Affiliations:** Mine Water and Environment Research Centre (MiWER), School of Science, Edith Cowan University, Joondalup, WA, Australia

**Keywords:** prokaryotic microorganisms, global data set, Anthropocene, research effort, ecological restoration, mine water

## Abstract

Pit lakes are one of the greatest legacies of open-cut mining. Despite the potential hazards of these lakes, they represent newly formed ecosystems with great scientific and ecological potential. Although thousands of pit lakes occur on every inhabited continent, with more being created, the microbial ecology of pit lakes is relatively under-researched. We evaluated the current state of microbial research in pit lakes by performing a Web of Science search and creating a literature database. Study lakes were categorized according to location and water quality (pH and conductivity) which is a key community and environmental concern. Research technology employed in the study was also categorized. We compared research effort in lakes, rivers, and streams which are the more “charismatic” inland aquatic ecosystems. Pit lake publications on microbes from 1987 to 2022 (*n* = 128) were underrepresented in the literature relative to rivers and streams (*n* = 321) and natural lakes (*n* = 948). Of the 128 pit lake publications, 28 were within the field of geochemistry using indirect measures of microbial activity. Most pit lake microbial research was conducted in a few acidic lakes in Germany due to social pressure for remediation and government initiative. Relatively few studies have capitalized on emerging technology. Pit lake microbial research likely lags other more charismatic ecosystems given that they are viewed as performing “ecosystem disservices,” but this is socially complex and requires further research. Improving understanding of microbial dynamics in pit lakes will allow scientists to deliver safer pit lakes to communities.

## Introduction

Mining is the backbone of civilization (Younger et al., 2002; Coulson, 2012), and mine-pit lakes (“pit lakes”) are created when open-cut mines are flooded with water at the end of mining operations. When first formed, pit lakes are often low in biodiversity and nutrients, spatially homogeneous, lack higher trophic levels, and contain little organic matter (Lund et al., 2020). Pit lakes may have poor water quality as a result of groundwater and surrounding geology (e.g., Younger and Wolkersdorfer, 2004; Falagán et al., 2014; Soni et al., 2014; Marszelewski et al., 2017; Sakellari et al., 2021). Poor water quality has caused public alarm (Woodbury, 1998; Kean, 2009; Robbins, 2016), although where pit lakes have been actively rehabilitated, they are spectacular examples of recreation and cultural renewal (Weber, 2020).

Charismatic ecosystems tend to receive more scientific and public attention (Duarte et al., 2008), and pit lakes are often perceived as non-charismatic waterbodies responsible for “ecosystem disservices” (e.g., Zeide, 1998; von Döhren and Haase, 2015; Blanco et al., 2019) mainly related to poor water quality and physical safety of the site (McCullough and Lund, 2006; Graupner, 2009; Mantero et al., 2020; Newman et al., 2020; Sánchez-España et al., 2020b; Rönicke et al., 2021). However, every pit lake is different, and if well managed and designed, some pit lakes could represent a “transitional ecosystem disservice” (Saunders and Luck, 2016) with future benefits for communities and the environment in terms of recreation, conservation, and industry (Blanchette and Lund, 2016; Palit and Kar, 2019; Williams et al., 2020). Pit lakes also provide scientists a platform from which to discover new species, metabolic functions, biogeochemical processes, and interactions between and within trophic levels, often in extreme environments (e.g., Falagán et al., 2014, 2017a; Falagán and Johnson, 2014, 2016). However, factors such as the environmental stigma surrounding pit lakes, a lack of interdisciplinary scientific collaboration, and unpublished data held by the mining industry have likely hindered scientific progress in the field of pit lake research relative to other inland aquatic ecosystems (Blanchette and Lund, 2020).

Although research on microbial taxonomic diversity in pit lakes has been limited, pit lakes host a diverse microbial community within their waters and sediments (Blanchette et al., 2020; Grettenberger et al., 2020; Blanchette and Lund, 2021; Xin et al., 2021). From our knowledge of other aquatic ecosystems, microbes will play a key role in pit lake ecosystem function through nutrient cycling, the fate and transport of metals and metalloids, mineral formation, biogeochemical processes, decomposition of organic matter, and interactions with other organisms (Oren, 2004; Pace et al., 2016; Gupta et al., 2017; Flemming and Wuertz, 2019; Morris et al., 2020). Despite the growing popularity of microbial diversity and function research in terrestrial and aquatic environments, less is known about the overall taxonomic and functional diversity of the microbial community and their roles in pit lakes.

This mini review synthesizes the current state-of-the-art on prokaryotic microbial research in pit lakes *via* a literature review and analysis. We also provide metrics on other inland water bodies (rivers and naturally formed lakes) and categorize the pit lakes in terms of pH and conductivity because water quality is a key concern for communities. We provide a synthesis on the most-researched lakes and technologies employed and how to advance the field. This research is important because understanding microbial function is critical for restoring and providing ecosystem services (Singh et al., 2019; Dutta and Sen, 2021). Improving the scope of microbial research in pit lakes will allow scientists to manage risks and provide benefits to catchments and communities.

## Methods

To determine the number of pit lake papers investigating microbes, a data set was created using Web of Science (WOS).^1^ Two broad pit lake literature searches were conducted on August 31, 2022 “Across all the databases” searching the “Topic field.” The first search method used a combination of 16 terms (i.e., “pit lake” AND “bacteria”): “pit lake (s),” “mine (lake (s)),” “Anthropocene (lake),” “(aquatic) ecosystem,” “bacteria,” “microbial community (ies),” “microbe (s),” “microbiome,” “16S,” “amplicon,” “sequencing,” “meteganomics,” “metaproteomics,” “metatrascriptomics,” “sulphate reducing bacteria,” and “SRB.” The second search method used the following searching strategy: (bacteria OR microbial community OR microbial communities OR microbe OR microbes OR microbiome OR 16S OR amplicon OR sequencing OR metagenomics OR metaproteomics OR metatrascriptomics OR sulphate reducing bacteria OR SRB OR microorganism) AND “pit lake” NOT (“crater lake” OR “bioreactor” OR “natural lake”). The second search method was conducted six times, changing the terms after the AND using the terms “pit lake (s)” (search one and two), “mining lake (s)” (search three and four), and “mine lake (ML) (s)” (search five and six). Additionally, if any papers were identified from reference lists of searched papers, they were added to the data set. Results from all searches were combined to form one data set (Supplementary Table 1). The dataset contains 221 data points extracted from 128 pit lakes microbial papers. Each data point refers to data extracted from each lake within a publication. Non-English papers, reviews, abstracts, meetings, books, and irrelevant papers were excluded from the data set. Tailing ponds were also excluded from the data set, although Base Mine Lake (BML, Alberta, Canada) was included. Compared to “common” tailing ponds, BML has a deeper freshwater and mine water “cap” (∼10 m vs. <5 m) over the tailings (Arriaga et al., 2019).

From each pit lake publication we extracted data related to the pit lake: country, lake name, latitude and longitude (decimal degrees), target resource, water pH, and conductivity. The research study was categorized in terms of broad microbial methodology (e.g., most probable number, sequencing) and type of sample collected (e.g., water, sediment, “iron snow”). Depending on the reported data, which may have been collected over time, environmental parameters such as pH and conductivity were extracted either as a single value or minimum and maximum values available. Pit lakes were classified according to pH as (a) acid (pH 1–6.4), (b) circumneutral (pH 6.5–7.5), (c) neutral (pH 7), or (d) alkaline (pH 7.6–14). Based on conductivity lakes were either (a) fresh (< 1499 uS/cm), (b) brackish (1500–1999 uS/cm), or (c) saline (> 2000 uS/cm). The salinity of most acidic pit lakes, particularly in the deeper anoxic layers, is predominantly caused by high concentration of SO_4_^2–^), Ca, Mg, Al, and dissolved iron (Wollmann et al., 2000; Falagán et al., 2016) rather than sodium chloride (NaCl) characteristic of seawater or saline lakes. Conductivity and pH of pit lakes included in the data set were summarized using a Sankey diagram.^2^

In order to compare the relative number of pit lake microbe papers to research in other inland water bodies, three separate literature searches for microbial research in lakes, rivers, or streams was conducted in WOS broadly following the pit lake search format across “All Databases” searching the topic field: (1) (bacteria OR microbial community OR microbial communities OR microbe OR microbes OR microbiome OR 16S OR amplicon OR sequencing OR metagenomics OR metatranscriptomics OR sulphate reducing bacteria OR SRB) AND lake*, (2) (bacteria OR microbial community OR microbial communities OR microbe OR microbes OR microbiome OR 16S OR amplicon OR sequencing OR metagenomics OR metatranscriptomics OR sulphate reducing bacteria OR SRB) AND river*, and (3) (bacteria OR microbial community OR microbial communities OR microbe OR microbes OR microbiome OR 16S OR amplicon OR sequencing OR metagenomics OR metatranscriptomics OR sulphate reducing bacteria OR SRB) AND stream*. The search was limited to papers published between 1987 and 2022 corresponding to the period of pit lake microbial research and results were refined as above. The following terms were excluded from the searches: “pit lake,” “ML,” “mining lake,” quarry lake, “floodplain lake,” “reservoir,” “cave river,” “karst river,” “estuary,” and “delta.” Due to the large volume of global research on inland water bodies and different local names for these ecosystems, we limited our search to the terms “lake,” “river,” and “stream.” Web of Science research areas included: “Environmental Sciences Ecology,” “Biochemistry Molecular Biology,” “Marine Freshwater Biology,” “Microbiology,” Life Sciences Biomedicine Other,” “Microscopy,” “Biodiversity Conservation,” “Water Resources,” and “Biotechnology Applied Microbiology.”

## Results and discussion

### Pit lakes are underrepresented in the research literature

Over the past 35 years (1987–2022) 687 papers on pit lakes have been published and 128 of these papers were microbial research (Supplementary Table 1). In the pit lake data base (*n* = 128 papers), 28 papers investigated geochemistry, which by nature inferred microbial activity through elemental and chemical analysis. Therefore, in the context of the search methodology, 100 published papers have directly measured microbial assemblage, activity, function, or density in pit lakes (Supplementary Table 1). The number of pit lake microbe papers per year between 1987 and 2022 ranged between zero and eight in 2021. During the same period, a greater number of papers on microbial research were published in rivers and streams (*n* = 321) and lakes (*n* = 948).

Our results support the idea that there is a positive relationship between research effort and ecosystem charisma (Duarte et al., 2008; He et al., 2021). Pit lake ecosystems are often associated with environmental stigma and “ecosystem disservices” such as poor water quality and visual disruptions in the landscape (Birkhofer et al., 2015; Lopez and Blanchette, 2020). It was surprising that research effort (measured in terms of “papers published”) conducted in rivers and streams was low relative to lakes, given that rivers were more often studied from 2000 to 2010 compared to lakes, wetlands, and ponds (Stendera et al., 2012). However, other researchers have found riverine microbe studies to be similarly underrepresented (de Oliveira and Margis, 2015), and aquatic microbes in general were underrepresented relative to other taxa such as fish and macroinvertebrates (Stendera et al., 2012). While determining the broad drivers of ecological research is outside the scope of this study, microbes may be considered non-charismatic taxa and therefore generally overlooked in terms of research (McGinlay et al., 2017).

In addition to a potentially biased perception of pit lake microbial ecology, the lack of published papers in this area may be due to more practical reasons. Pit lakes are created through mining, and “data loss” in industry reports or unpublished student research, as well as a lack of collaboration between industry and scientists of different disciplines are bottlenecks to research publication (Blanchette and Lund, 2020). Further, freshwater ecologists and microbiologists are not exposed to pit lake ecosystems (sensu Duarte et al., 2008; He et al., 2021), often because pit lake microbial research is published in “specialized” journals (e.g., Aquatic Geochemistry, Geobiology, Mine Water and the Environment, and Hydrobiologia) and thus unlikely to reach a broad microbiologist audience. However, given the advancement and adoption of new technology (e.g., “-omics”) and increased collaboration, microbial research in pit lakes is an emerging field with great potential.

### Pit lake research is “downstream” of population

Despite the presence of thousands of pit lakes on all inhabited continents (Castendyk and Eary, 2009), most pit lake microbial research (*n* = 128 papers, Supplementary Table 1), has been conducted in Germany (*n* = 60 papers) and Spain (*n* = 29 papers). Fewer studies were conducted in Canada (*n* = 13 papers), Australia (*n* = 7 papers), and the United States (*n* = 4 papers). The 128 microbial papers yielded “data points” (*n* = 221) from 99 pit lakes as different authors sampled the same pit lake (Supplementary Table 1). Of the 221 data points, 63 were extracted from German pit lakes, particularly ML 111 (*n* = 39), ML 77 (*n* = 14), and ML 117, (*n* = 10), followed by the Spanish Iberian Pyrite Belt (IPB) lakes Cueva de la Mora (*n* = 15) and Guadiana (*n* = 9).

Pit lake research in Germany and Spain has been driven by geography and water quality which is underpinned by political and social demand (Weber, 2020). In Germany, pit lake rehabilitation and research were national priorities after reunification (Benthaus et al., 2020). The nationally owned company Lausitzer und Mitteldeutsche Bergbau-Verwaltungsgesellschaft (LMBV) gained control of the flooded lignite mines in populated areas and was tasked with remediating East Germany’s acidic pit lakes (Benthaus et al., 2020), specifically Mine Lake 111 in Lusatia (East Germany) (Karakas et al., 2003; Kleinsteuber et al., 2008). The social, political, and environmental investments in German pit lake remediation have established and driven global pit lake research.

Although the microbiology of Spain’s pit lakes (e.g., Guadiana and Cueva de la Mora) are the second-most studied relative to Germany’s, they have undergone less research and remediation likely due in part to their remote locations (Sánchez-España, pers. comm.). Therefore, social pressure was less important in Spanish pit lake research than in Germany due to population density, with Spanish pit lake microbial research largely driven by the interests of scientists (Sánchez-España, pers. comm.). Further, many Spanish pit lakes are located on private active mine sites, meaning that unlike in Germany, the Spanish government had no broad legal authorization to conduct remediation or research, with some lakes eventually being drained and re-mined (Sánchez-España, pers. comm.). Mining is a key feature of the Anthropocene, and it follows that pit lake research would also be driven by social and population dynamics.

### Low pH drives pit lake research

**FIGURE 1:**
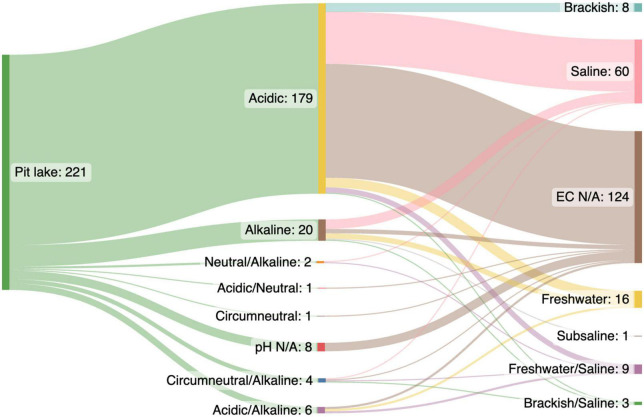
Water quality (pH and conductivity) of 221 data points extracted from a Web of Science (WOS) literature search (*n* = 128 papers) for research on pit lake microbiology (Supplementary Table 1). Most data points (*n* = 179) were extracted from acidic pit lakes (pH < 6.4) vs. circumneutral, neutral, and alkaline pit lakes (pH > 6.5; *n* = 27 data points).

Most microbiological research in pit lakes has been conducted in acidic lakes (pH < 6.5; *n* = 179 data points), with fewer data points from alkaline (pH = > 7.5; *n* = 20), and circumneutral (pH = 6.5–7.5; *n* = 1) pit lakes (Figure 1). Acidic pit lakes form in abandoned coal, gold, base metal, and uranium pits due to a combination of factors such as the exposure of sulphides and minerals to water and oxygen (van der Graaf et al., 2020), an absence of neutralizing carbonates, groundwater chemistry, and the activity of sulphur and iron-oxidizing bacteria (e.g., Castro and Moore, 2000; Frömmichen et al., 2003; González-Toril et al., 2013). Often, acidic pit lakes are characterized by high concentrations of dissolved metals (e.g., iron and aluminium), sulphate (SO_4_^2–^), and ammonium (NH_4_^+^), and low concentrations of nitrate, phosphorous, and organic carbon (Castro and Moore, 2000; Soni et al., 2014; Lund and McCullough, 2015). As a result, acidic pit lakes generally contain low levels of primary production and have microbial communities dominated by extremophiles involved in the cycling of sulphur and iron (Kamjunke et al., 2005; Fyson et al., 2006; Wendt-Potthoff et al., 2011).

Acidic pit lakes and associated mines are considered hostile environments posing potential threats for wildlife and humans (McCullough and Lund, 2006; Hadjipanagiotou et al., 2020; Sergeant et al., 2022). Unsurprisingly, understanding and rehabilitating these lakes has become a priority for industry, government agencies and consequently researchers (Meier et al., 2004; Gammons and Icopini, 2020). Although abiotic approaches such as limestone addition and flooding with alkaline water have been used successfully (e.g., Neil et al., 2009; Benthaus et al., 2020), many rehabilitation strategies have focused on microbially mediated neutralization processes (e.g., Geller et al., 2009).

Pit lake microbial research has been dominated by laboratory studies conducted at micro- and mesocosm scales to determine what active interventions may improve pit lake water quality (e.g., Castro and Moore, 2000; Costa and Duarte, 2005; McCullough et al., 2006; Mccullough and Lund, 2008; Kumar et al., 2011; McCullough and Lund, 2011; Lund and McCullough, 2015; Falagán et al., 2017b). These remediation studies mainly focused on the presumed water quality effects of specific microbial groups and processes such as iron- or sulphur-reducing bacteria or dissimilatory reduction (Fauville et al., 2004; Costa and Duarte, 2005), rather than the microbial ecology of pit lakes.

Most data points (*n* = 124) did not report conductivity data for pit lake studied. The remaining 97 data points were predominantly saline (>2000 uS/cm, *n* = 60) (Figure 1). Saline lakes were also likely to be acidic (pH < 6.4) (Figure 1) and therefore salinity was probably due to high concentrations of SO_4_^2–^, Fe (II), Ca, and Mg (e.g., Wollmann et al., 2000; Falagán et al., 2016) rather than NaCl. In contrast to acidic pit lakes, non-acidic (pH > 6.5) moderately saline pit lakes are often considered benign environments with low rehabilitation priority. Thus, like natural saline lakes (Williams, 1996), saline pit lakes are under-researched (Williams, 1996; Mori et al., 2019; Blanchette and Lund, 2021). Similar to acid pit lakes, saline pit lake rehabilitation strategies would focus on water quality improvement or minimization of pit outflows (Mehanna et al., 2010; Mouhamad et al., 2017; Nielsen, 2020). Natural saline lakes provide ecosystem services such as minerals, water, habitats, aquaculture, tourism, and recreational activities (Williams, 1996; Wurtsbaugh et al., 2017; Edwards and Null, 2019). Similar services may be found in saline pit lakes, although this may require changing public perception (Lopez and Blanchette, 2020; Rosa et al., 2020; Svobodova et al., 2021).

Until recently, pit lake microbial science was slow to capitalize on emerging technology (Supplementary Table 1). Many papers in our data base used most probable number to determine the concentration of specific microbial groups (Kumar et al., 2011; Wendt-Potthoff et al., 2011, Koschorreck and Wendt-Potthoff, 2012). Community composition was often determined using gradient gel electrophoresis (DGGE) or terminal restriction fragment length polymorphism (T-FRLP) which limit the detection of rare taxa (<1% of the total abundance) (Kampe et al., 2010; Falagán et al., 2013; González-Toril et al., 2013). Although only a few studies have been conducted on metatrascriptomics, metagenomics, and metaproteomics in pit lakes (White et al., 2015; Mori et al., 2019; Ayala-Muñoz et al., 2020, 2022; Sánchez-España et al., 2020a; Ayala-Muñoz et al., 2022) papers utilizing emerging technology have introduced the field of pit lake microbial research to the wider microbiological community.

## Conclusion: Advancing the field of pit lake microbial research

**FIGURE 2:**
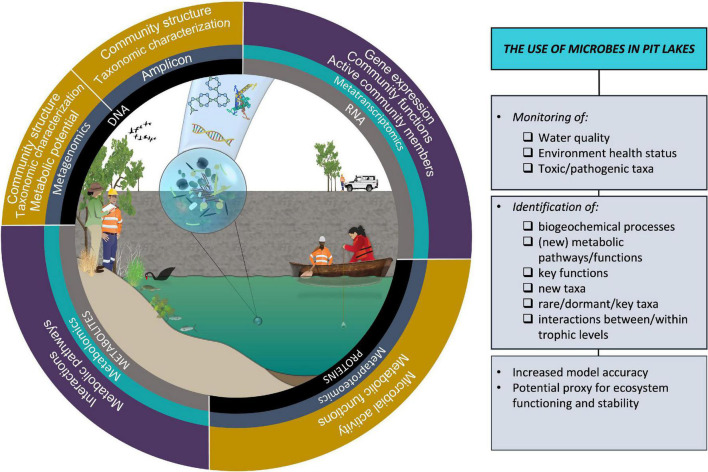
Microbes can be used in pit lakes for environmental monitoring and identification of processes and taxa. Harnessing new technology will advance the field of pit lake microbiology. Symbols curtesy of Integration and Application Network (ian.umces.edu/symbols/) and NESP Resilient Landscapes Hub (nesplandscapes.edu.au).

Pit lakes provide a unique opportunity to investigate colonization, succession, temporal changes of newly formed ecosystems, and novel microbial metabolic pathways (Kampe et al., 2010; Marszelewski et al., 2017, Sánchez-España et al., 2018) (Figure 2). Like any ecosystem, pit lakes host a rare microbial biosphere that plays key roles in nutrient cycling, pollutant degradation, and protection from pathogens (Pascoal et al., 2021) and hosts unique extremophile organisms (Stierle et al., 2006). However, we found that pit lake microbial papers were underrepresented in the literature relative to rivers/streams and lakes. This may have been due to the correlation between research effort and ecosystem charisma given the environmental stigma and ecosystem disservices associated with pit lakes (real and perceived), or for more practical reasons given that pit lake research has been traditionally performed by industry. We also were unable to include non-English papers, which may have underrepresented actual research effort. Regardless, pit lake research has been driven by population and therefore social pressure for remediation in acidic lakes as seen in Germany, which has produced the bulk of papers on pit lake microbiology. Advancing the field of pit lake microbiology will require capitalizing on new technology and publishing in broad microbiological journals in order to promote interdisciplinary and international collaboration. With thousands of pit lakes on all inhabited continents, raising the profile of this research area will benefit communities and the environment.

## Author contributions

All authors conceptualized, wrote the manuscript, contributed to the article, and approved the submitted version.

## Footnotes

1
https://clarivate.com/webofsciencegroup/solutions/web-of-science/

2
http://sankeymatic.com/build/
